# Oxytocin can decrease germ cells apoptotic index in testis under acute ischemia reperfusion in a rat model

**Published:** 2015-05

**Authors:** Rezvaneh Ghasemnezhad, Fahime Mohammadghasemi, Masoumeh Faghani, Mohammad Hadi Bahadori

**Affiliations:** 1 *Department of Anatomy, Guilan University of Medical Sciences, Rasht, Iran.*; 2 *Cellular and Molecular Research Center, Guilan University of Medical Sciences, Rasht, Iran.*

**Keywords:** *Oxytocin*, *Ischemia reperfusion injury*, *Testis*, *Apoptosis*

## Abstract

Ischemia reperfusion (IR) is the main pathology of torsion of testis and it is a common urologic emergency. There is some evidence that shows oxytocin (OT) plays role in ischemia reperfusion.

**Objective:**

To evaluate this hypothesis that OT can decrease germ cell apoptotic index in testis under acute ischemia reperfusion in a rat model.

**Materials and Methods:**

20 adult rats were randomly divided into four groups: Control, IR, OT and IR+ OT (OTA). Testicular ischemia was achieved by 720° torsion of the left testis for 2 hr. Then, torsion was removed and reperfusion was performed. Immediately after induction of reperfusion 0.03 µg/kg OT were administered intraperitoneally to the IR+ OT. Three hours after surgery left testis was removed and evaluations were made by Johnson’s score, ELISA, immunohistochemistry and histomorphometry for study of maturity of spermatogenesis, endocrine profiles, apoptosis and quantitative studies, respectively.

**Results:**

The results showed in addition tissue edema and congestion, a significant reduced in Johnson’s score were detected in IR group in comparison with controls (p=0.01), and apoptotic index increased significantly (p=0.001). Administration of OT in OT+IR group, increased Johnson’s score but it was not statistically significant. Germinal epithelium thickness was increased significantly (p=0.03), although apoptotic index decreased significantly in comparison with the IR group (p=0.04). However there was not significant difference in serum levels of testosterone, FSH and LH in none of groups (p=0.07).

**Conclusion:**

These results suggested that OT can decrease apoptotic index and improves complication of acute ischemic reperfusion in testis in a rat model.

## Introduction

Testicular torsion or, more correctly torsion of spermatic cord, is a common urologic emergency occurring in male newborns, children, and adolescents (1). Its incidence is about 1 in 4000 males by the age of 25 years (2, 3). Rapid diagnosis and immediately surgical treatment is necessary to avoid permanent testicular damage (4). The main pathophysiology of testicular torsion-detorsion is ischemia-reperfusion (IR) injury of the testis, and it causes over generation of reactive oxygen species that leading to cell membrane lipids peroxidation, protein denaturation and DNA damage, cell dysfunction and finally death or apoptosis (3, 5). The adverse effect of IR injury in tissues such as liver, brain, kidney, heart and stomach have already been reported (6-12). Oxytocin (OT) is a small Nano peptide synthesized in the Para ventricular and supraoptical nuclei of the hypothalamus, OT is also produced in some tissues such as heart, uterus, placenta, amnion, corpus luteum, and testis (13, 14). In addition OT has been considered to be a cardiovascular hormone produces and releases by the heart and large vessels (15).

Traditionally it was shown as a female hormone due to its role in parturition and milk ejection, but some researches have been shown its roles in male reproduction, increasing testosterone production by Leydig cells and spermiation (16-18). Some studies have indicated that OT has protective effect on IR injury in heart, kidney, stomach and urinary bladder (9, 12, 19, 20). The anti-apoptotic effect of OT, excepting in some study in some tissue such as heart and ovary is available (21, 22). Our review of the literature did not reveal any studies that investigate the anti-apoptotic effects of OT on testis tissue. Therefore we used OT in testis IR injury for the first time and the purpose of this study was determination of this hypothesis that OT can decrease apoptotic index in testis under acute ischemic reperfusion.

## Materials and methods

**Animals and surgical procedure**

This experimental study has been carried out in Guilan University of Medical Sciences. Twenty male Wistar Albino rats weighting (280-330 gr) were used in this study. The animals were maintained on a constant 12 hr light/dark cycle at constant room temperature (23±2^o^C) and humidity (60-70%) ambient and with free accesses to rat chow and tab water. All experimental procedures were done according to the guidelines of Animal Ethical Committee of Guilan University of Medical Sciences. Animals were randomly divided into four groups: Control, IR, OT and IR+OT. Surgery was conducted under intraperitoneally injection of ketamine (50 mg/kg) and Xylazine (2.20 mg/kg) anesthesia (23). All procedures were performed under sterile condition. In control group left testis was brought out through a median scrotal incision. In the IR group left testis was exposed through the same incision then left testis was rotated 720 in a clockwise direction and was maintained in this position by ligating of spermatic cord using 02 silk sutures for 2 hr, in order to prevent drying of the tissue exposed to the air, cotton soaked in saline was used (2).

After 2 hr testis was counter-rotated to the natural position consequently reperfusion was induced. In OT+IR group the same surgical procedure was performed as in IR group, OT (0.03 µg/kg; Sigma Chemical company) was injected intraperitoneally immediately after induction of reperfusion (21, 24). In OT group animals just received OT (0.03 µg/kg) via IP injection. Orchiectomy was performed 1 hr after reperfusion induced. The testes were fixed in 10% neutral buffered formalin, dehydrated, and embedded in paraffin. Then, 5-µm sections were prepared, and at least 5 slides from each testis were stained with H-E for histological assessment. Light microscopy was used for the evaluations.

**Spermatogenesis assessment**

The quality of spermatogenesis in each seminiferous tubule was scored according to Johnson’s scoring method (25). This method applies a grade from 1-10 to each tubule cross section according to following criteria: 10) complete spermatogenesis; 9) many spermatozoa present but disorganized spermatogenesis; 8) only a few spermatozoa present; 7) no spermatozoa but many spermatids present; 6) only a few spermatids present; 5) no spermatozoa or spermatids present but many spermatocytes present; 4) only a few spermatocytes present; 3) only spermatogonia present; 2) no germ cells present; and 1) no germ cells or Sertoli cells present. 10 seminiferous tubule cross sections were observed for each animal and a mean score count was tabulated.

**Morphometric study of spermatogenesis**

Morphometric study of seminiferous tubules was done by using a graded microscopic lens. For this purpose the thickness of germinal epithelium and diameter of lumen and seminiferous tubule were estimated in 20 transverse epithelium in each animal. The number of A spermatogonia, pachytene and leptotene primary spermatocytes, round spermatids and elongated spermatids were counted in 20 tubule in area of 1×1 mm^2^ (25, 26).

**Hormone measurement**

Blood samples were collected through the inferior vena cava, immediately after sacrificing the rat. The serum was separated and stored at -80^o^C. Serum testosterone and luteinizing hormone level concentrations were measured using ELISA kits (Demeditec Diagnostics GmbH, Germany).

**Evaluation of germ cell apoptosis**

Germ cell apoptosis was evaluated by TUNEL [terminal deoxynucleotidyl transferase enzyme mediated dUTP nick end labeling] according to the instructions in the TUNEL assay kit (Roche Germany). Briefly, serial 5 µm thick paraffin-embedded sections were deparaffinized with xylene (20 min) and rehydrated in graded alcohol (each 2 min), then they were incubated with 1 µl proteinase K (20 min) and washed tree time with phosphate-buffered saline after this, they were incubated with 5 µl TUNEL solution (40 min), and washed three time with phosphate-buffered saline, incubated with 10 µl pod-convertor (30 min), and washed three time with phosphate-buffered saline, and finally when sections were incubated with 10 µl DAB substrate (60 min) and washed with distilled water, counter stain staining was performed with Hematoxylin and then dehydrate in grade alcohol clear and dark brown cells were considered as apoptotic seminiferous tubules were estimated in 20 transverse epithelium in each animal and apoptotic index was determined by the formula 100× (number of TUNEL positive cells nuclei/total number of cell nuclei) (27).

**Statistical analysis**

The data were analyzed by the analysis of variance (ANOVA) and Tukey post Hoc tests. The p<0.05 was considered statistically significant.

## Results

**Histological examination**

**Figure 1:**
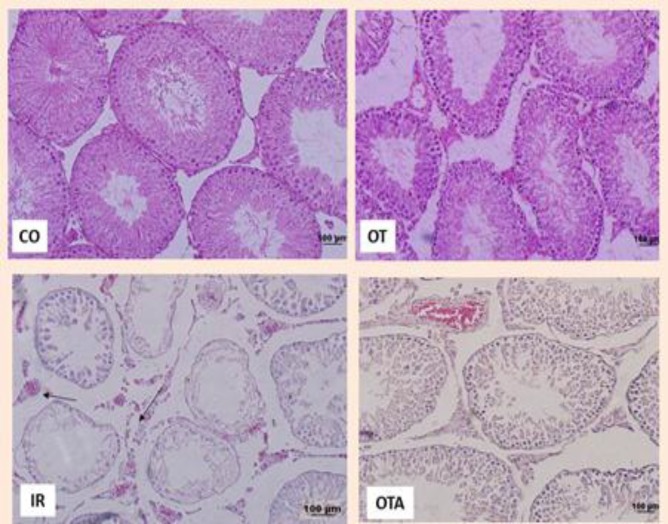
Photography of seminiferous tubules. Arrow point to edema and congestion H&E staining ×200

Figure 1, table I and table II demonstrate the histological findings and mean of Johnson’s score of each group in testis and morphometric measurement respectively. Based on light microscopy observation in control group, spermatogenesis was active and all of germ cells line were seen, lumen had a regular border and abundant mature spermatozoids and Sertoli cells were observed. In addition in interstitial tissue blood vessels, connective tissue cells and Leydig cells were seen. OT group had no morphologically different with control group while in IR group edema and blood vessels congestion were seen, there was a marked decrease in the seminiferous tubular diameter and germinal layer thickness and Johnson score in IR group (p=0.01).

In OT+IR group edema and blood vessels congestion improved and Johnson score increased morphologically but no statically significant. Even though the thickness of germinal epithelium increased significantly (p=0.03) but there wasn’t significant difference in tubular diameter (Table I). Table II shows the number of cells, our results showed that germ cells number except pachyten cells had not significant statistical difference in each groups so that in IR group cells number had no significant difference in camparision with control while pachyten cells had a significant decrease (p=0.0001). However administration of 0.03 µg/kg OT immediately after reperfusion induction led to significant increase in pachyten cells number (p=0.04).

**Hormone measurement**

Table III shows serum levels of endocrine profiles serum levels of testosterone, FSH and LH were in normal range as expected in control and OT group. Also there was no significant difference in serum levels of testosterone, FSH and LH in IR and IR+OT groups (p=0.07).

**Apoptosis analysis**

**Figure 2:**
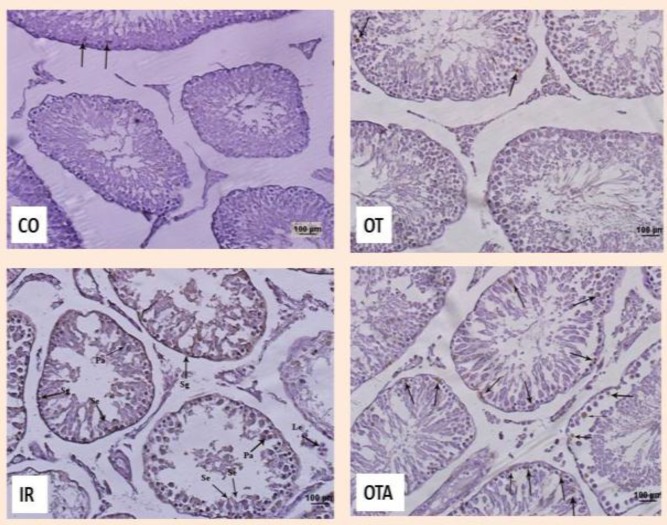
Photography of seminiferous tubules. Arrows point to apoptotic cells TUNEL staining ×200

**Figure 3:**
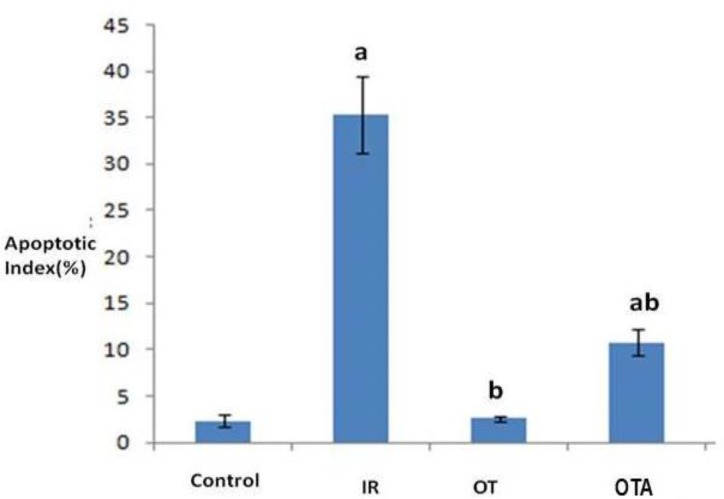
Graph shows Ischemia-reperfusion injury and oxytocin effect on germ cell apoptosis index in adult rat testis.

Evaluation of germ cell apoptosis showed that apoptotic cells were seldom in both of control and OT groups and apoptotic index was not significant as is shown in figure 2 but in IR group apoptotic cell were abundant and apoptotic was increased significantly statically (p=0.001), while in IR+OT group, number of apoptotic cell decreased and apoptotic index decreased significantly in comparison with IR group (p=0.04) (Figure 2, Figure 3).

**Table I:** Ischemia-reperfusion injury and effect of oxytocin on morphometric parameters and Johnson score of the adult rat testis

**Group**	**Tables diameter**	**Germinal layer thickness**	**Duct diameter**	**Johnson score**
CO	55.305 ± 55.30b*	1.159 ± 33.35b*	82.6 ± 8.0	6.9 ± 8.1b*
IR	237.95 ± 0.35a*	85.74 ± 95.5a*	8.123 ± 8.25a*	94.7 ± 11a*
OT	75.299 ± 68.20	6.108 ± 55.3	20.145 ± 41.25a*	14.9 ± 26b*
OTA	15.293 ± 58.40	9.135 ± 43.50	55.154 ± 0.54a*	54.8 ± 39

All data are expressed as mean±SD.

ANOVA and Tukey post Hoc tests

CO: Control

IR: Ischemia-reperfusion

OT: Oxytocin

OTA: Oxytocin+IR

a* : Statistically significant compared with the control group (p=0.01). Johnson score column statistically significant compared with the control group (p=0.01).

b* : Significant compared to the IR group (p=0.01).\

**Table II:** Ischemia-reperfusion injury and oxytocin effects on adult rat testicular germ cell count

**Group**	**Spermatogonia**	**Preleptoten**	**Pachyten**	**Round spermatid**	**Elongated spermatid**	**p-value**
CO	1.66 ± 0.27	9.3 ± 1.53	11.69 ± 1.50	38.62 ± 3.08	26.43 ± 4.40	p>0.05
IR	1.60 ± 0.54	8.38 ± 1.95	7.46 ± 1.78a*	31.16 ± 3.31	20.69 ± 11.97	p=0.0001 vs. p>0.05
OT	1.54 ± 0.37	8.55 ± 2.48	9.82 ± 1.13	35.26 ± 3.31	21.41 ± 8.12	p>0.05
OTA	1.55 ± 0.44	8.87 ± 2.18	7.18 ± 1.54b*	35.06 ± 3	21.20 ± 8.63	p=0.04vs p>0.05

All data are expressed as mean±SD.

ANOVA and Tukey post Hoc tests

CO: control

IR: ischemia-reperfusion

OT: Oxytocin

OTA: Oxytocin+IR

a* : Significant in compare whit the control group (p=0.0001).

b* : Significant in compare whit IR (p=0.04).

**Table III:** Ischemia-reperfusion injury and oxytocin effect on endocrine profiles adult male rats

**Group**	**Testosterone (ng/ml)**	**Luteinizing hormone (lu/l)**	**Follicle stimulating hormone (mlu/l)**
CO	3.26 ± 0.19	3.66 ± 0.29	239.08 ± 9.05
OT	2.54 ± 0.79	3.32 ± 0.52	230.42 ± 7.55
IR	3.09 ± 0.22	3.45 ± 0.45	263.93 ± 7.53
OTA	2.94 ± 0.78	3.49 ± 0.58	321.45 ± 4.10

All data are expressed as mean±SD.

ANOVA and Tukey post Hoc tests (p=0.07)

CO: Control

IR: Ischemia-reperfusion

OT: Oxytocin

OTA: Oxytocin+IR

CO: Control

OT: Oxytocin

IR: Ischemia-reperfusion

OTA: Oxytocin+IR

CO: Control

OT: Oxytocin

IR: Ischemia- reperfusion

OTA: Oxytocin+IR

All data are expressed as mean±SD.

ANOVA and Tukey post Hoc tests

CO: Control

IR: Ischemia-reperfusion

OT: Oxytocin

OT+IR: Oxytocin+IR.

a: Significant compared to the control group (p=0.001)

b: Significant compared to the IR group (p=0.04).

## Discussion

The main pathophysiology of testicular torsion is ischemia-reperfusion that leads to testicular damage, apoptosis and finally infertility. The histological damage of IR injury in testis has been shown in many studies with different time and degree of torsion and different time of reperfusion (3-6). IR injury by several mechanisms including increased ROS, production and release of inflammatory factors such as the release of apoptotic caspases cascade enzymes promotes a decrease in blood flow can cause tissue damage (28). Previous research has shown that the severity of ischemic tissue damage associated with the duration and degree of twist (29).

Kurcer and colleagues in a study that ischemia was performed for 1 hr and reperfusion injury was induced for 24 hr, showed that this time of ischemia and reperfusion can cause testicular tissue damage (30). Filho *et al* in a study conducted in 2012, found that ischemia and reperfusion Injury for 1 hr creates some results similar to previous research (31). Although It has been shown that 720 ischemia is sufficient cause to cessation of the testicular blood flow, and its minimum time lead to testicular damage and germ cell apoptosis in a rat model (32). The adverse effect of IR injury in many tissue such as liver, heart have been shown (6, 33, 34). According to previous studies mentioned above in the present study, the ischemia time was chosen as, 2 hr with 1 hr reperfusion subsequently. furthermore our study showed that 720 ischemia for 2 hr and consecutive reperfusion for 1 hr led to edema and blood vessels congestion and histologically damage such as degeneration of germ cells layer and decrease germinal and luminal diameters, decreased Johnson’s score and number of germ cells especially pachytene cells and increase apoptotic index parallel with previous studies (1-3).

Spermatogenesis is a highly regulated process that is controlled mainly by the testosterone and gonadotropins (26). In this study there was not significant statistical difference in serum levels of testosterone, FSH and LH. Testosterone level reduced in IR group however it was not significant. There are conflicting reports about hormonal changes after testicular IR: in a prospective study in 2009, 5 years after detorsion treatment, The endocrine profiles were resistance to ischemia (35). In another study it was reported that the level of testosterone, luteinizing hormone, follicle stimulating hormone were normal with ischemia for 7 hr and 48 hr (36). Testicular hormonal functions partly well conserved except in patients with ischemia more than 8 hr or testicular atrophy (37). While reduction in testicular androgen production results in minimal duration and degree of ischemia in long term was reported As well (38). As previously mentioned, in our study endocrine profiles were not affected in none of groups (p=0.07).

Apoptosis or programed cell death that occurs in both physiologic and pathologic periods and naturally wave of apoptosis in testes of prepubertal mammals occurs, It appears that apoptosis is essential for the development of spermatogenesis in adolescence. Also in the adult testis spermatogenesis and spontaneous degeneration of germ cells is that they seem to have the highest amount of apoptosis leading to the loss of 75% of the cells mature spermatozoa (39). About 10 min after apoptosis induction Bcl2 proteins and mitochondria leads to cell death is initiated and in this phase of course different cells are at different intervals (40).

The role of apoptosis has been highlighted in testis ischemia-reperfusion (40). Furthermore the role of IR injury in apoptosis in tissues such as kidney and heart was reported (10). Although some studies reported the role of reperfusion in apoptosis and confirm that ischemia starts apoptosis and necrosis, reperfusion accelerates apoptosis. In this research we found a lot of TUNEL positive cells in group with 720 ischemia for 2 hr and consecutive reperfusion for 1 hr although statistical analysis confirmed an increase in apoptotic index in IR group compared to control group (p<0.001). OT is a small Nano peptide that is well known for its role in reproduction (16, 41). OT receptors are on the central nervous system, myometrium and uterine endometrium, heart, liver, kidney, myo-epithelial cells of the breast tissue and testis (12).

Steroid factors, especially estrogen have effects on OT and increases binding OT to the OT receptors. Although OT produced in testes and Leydig cells are responsible for that and LH has effects on hormone secretion. Furthermore OT is involved in the contraction of the seminiferous and epididymis duct (42, 43). Tugtepe and colleagues in 2007 reported protective effects of OT on apoptosis in kidney under ischemia-reperfusion injury, Erkanli and colleagues also evaluated protective effect of exogenous OT in skeletal muscle under ischemia-reperfusion and reported anti- apoptotic effects of OT before and during ischemia (9, 44).

Alizadeh *et al* during several studies that examined the effect of OT on ischemia- reperfusion, reported anti-inflammatory and anti-apoptotic effects of antioxidants (21, 24, 45). Although it was reported that OT can improve left ventricle ejection fraction in rats subjected to MI via intrinsic cardiac cholinergic neurons and NO release (46). Recently it have been shown that anti-oxidant can improve complication due to IR injury (1, 2). Antioxidant role of OT in IR injury in some tissue such as urinary bladder and skeletal muscle has been shown, although OT reduces IR injury in rat kidney, with improved renal function, via decreased serum creatinine and BUN levels in addition improved antioxidant status and reactive oxygen species (7, 18, 34).

Furthermore It was shown that OT same as other G-protein coupled ligands can act in such a way that PI3K/AKT activation and release onto downstream kinases, such as glycogen synthase kinase-3 beta, targeting the mitochondria to protect cells (44). Results of our study showed that administration of 0.03 µg/kg OT immediately after reperfusion induction decreases edema, blood congestion and increases Johnson’s score morphologically but the difference was not statistically significant, also this amount of OT improved germinal layer thickness and number of germ cells but it wasn’t significant statically. Anti-apoptotic effect of OT in IR injury previously has been shown in some tissues such as heart ovary and according to our knowledge this study is the first report of OT effect on apoptosis in IR injury in testis a rat model.

Based on light microscopy observation an obvious reduction in tissue edema and blood vessels congestion were seen and apoptotic cells were reduced in group OT+IR group in comparison with IR group and apoptotic index decreased significant statically (p<0.04), and this results confirmed that OT can decrease apoptotic index after acute ischemia reperfusion.

## Conclusion

In conclusion, we demonstrated the protective effect of OT on apoptosis immediately after reperfusion induction in testis for the first time and we can say that OT could improve spermatogenesis after IR injury and decrease apoptotic index without effect on LH, FSH and testosterone levels. We propose that OT may be a novel approach for improvement of testicular ischemia-reperfusion injury complication.

## Note

***This article extracted from M.Sc. thesis. (Rezvane Ghasemnezhad)***
